# Novel Use of Low-Dose Radiotherapy to Modulate the Tumor Microenvironment of Liver Metastases

**DOI:** 10.3389/fimmu.2021.812210

**Published:** 2021-12-15

**Authors:** Kewen He, Hampartsoum B. Barsoumian, Genevieve Bertolet, Vivek Verma, Carola Leuschner, Eugene J. Koay, Ethan B. Ludmir, Ethan Hsu, Esha Pisipati, Tiffany A. Voss, Nahum Puebla-Osorio, Maria Angelica Cortez, James W. Welsh

**Affiliations:** ^1^ Department of Radiation Oncology, Shandong Cancer Hospital and Institute, Shandong First Medical University and Shandong Academy of Medical Sciences, Jinan, China; ^2^ Cheeloo College of Medicine, Shandong University, Jinan, China; ^3^ Department of Radiation Oncology, The University of Texas MD Anderson Cancer Center, Houston, TX, United States; ^4^ Department of Radiation Oncology, Allegheny General Hospital, Pittsburgh, PA, United States; ^5^ Department of Gastrointestinal Radiation Oncology, The University of Texas MD Anderson Cancer Center, Houston, TX, United States; ^6^ Department of Biostatistics, The University of Texas MD Anderson Cancer Center, Houston, TX, United States

**Keywords:** low dose radiation, radiotherapy, immunotherapy, liver cancer, stroma

## Abstract

Despite multiple therapeutic approaches, the presence of liver metastases carries a guarded prognosis, urgently necessitating further clinical and scientific research to develop curative interventions. The liver is an immunoprivileged organ that suppresses the effectiveness of immunotherapies in patients with hepatic metastases. Cancer immunotherapies have been successfully bolstered by low-dose radiotherapy (LDRT), which is capable of reprogramming the tumor microenvironment (TME) from an immunosuppressive to an immunostimulatory one. Likewise, LDRT may be able to revoke the immune privilege enjoyed by the liver, permitting successful immunotherapies there. Here, we first review challenges that face the treatment of liver metastases. We next outline emerging preclinical and clinical evidence supporting enhanced systemic tumor control of LDRT in the context of cancer immunotherapy. Finally, we will discuss the rationale of combining liver-directed LDRT with immunostimulatory strategies to overcome immune resistance and achieve better clinical response. This notion is supported by a recent case study in which a patient who had progressed following T cell therapy experienced a complete response after LDRT to the liver.

## Introduction

Most cancers in the liver develop from colonizing metastases, rather than primary malignancies. Up to 50% of patients with various cancer diagnoses develop liver metastasis during the course of their disease (1). This is partly because the liver is associated with a unique “dual vascular supply”, comprising venous supply from abdominal tissue, as well as systemic arterial supply originating from extra-abdominal tissues. Of the numerous possible locations throughout the body for cancer to metastasize, it has been estimated that the liver accounts for roughly a quarter (2). Indeed, liver metastases are common for multiple solid tumors, and rates of liver metastases can vary from one tumor to another. For example, rates of liver metastases are high for uveal melanoma (~50%) (3, 4), colorectal cancer (30-50%), pancreatic adenocarcinoma (30-40%), and neuroendocrine tumors (20-46%); whereas cutaneous melanoma (10-20%), lung (4-17%), breast (6-38%), and gastrointestinal stromal tumors (5-40%) (1, 5) have more variable and generally lower rates of liver metastases.

A multitude of therapeutic approaches have been levied in attempt to improve the outcomes of liver metastasis patients. Recently, several preclinical and clinical studies (6–11) reported the outstanding safety, efficacy, and/or relating mechanisms of a novel non-ablative treatment termed low-dose radiotherapy (LDRT), which is defined as radiotherapy of 0.5-2 Gray (Gy) per fraction for up to 1-10 Gy total. These promising findings provide the rationale for the development of LDRT as a potential treatment alternative for patients with liver metastases.

## Clinical Treatment of Liver Metastases

In an effort to address multiple ongoing questions regarding therapy for liver metastases, we have summarized the results of a systematic search of clinicaltrials.gov for enrolling randomized trials for patients with liver metastases (**Supplementary Table 1**). For oligometastases in the liver (up to 3 lesions) (12), others have also evaluated a variety of local therapy options (1, 13, 14). These include but are not limited to: surgical resection; embolization by means of chemotherapy (e.g. trans-arterial chemoembolization [TACE]) or radionuclides (e.g. yttrium-90); hepatic artery chemotherapy infusion; immune embolization techniques; fractionated or stereotactic external beam radiotherapy (SBRT); or other ablative procedures (e.g. radiofrequency, microwave, or cryoablation). However, once the cancer reaches a polymetastatic state, such aggressive local therapeutic techniques can no longer be employed due to the damage they cause to the liver at that scale. Whole-liver RT or partial-liver RT has been shown to effectively palliate such patients, thereby improving quality of life (15–17), but may often lead to radiation-induced liver disease and may generate an unfurling hydra of complications that are difficult to manage (18).

Although checkpoint inhibition (CPI), adoptive cell therapy (ACT), and other immunotherapies have shown significant clinical benefit in patients with extrahepatic tumors, patients with metastases to the liver had been historically identified to respond poorly to immunotherapy (19, 20). For example, a sub-analysis of two phase-III trials, demonstrated decreased overall survival (OS) in non-small cell lung cancer (NSCLC) patients with liver metastases treated with nivolumab compared to the overall pooled population treated with nivolumab (3-year OS: 17% *vs*. 8%; median OS: 11.1 *vs*. 6.8 months, respectively) (21). In a phase-II trial conducted at MD Anderson Cancer Center, we found that response rates in non-irradiated tumors were 31% for lung versus 14% for liver metastases (*P*<0.061) (22). Similarly, an unpublished *post-hoc* analysis of a randomized phase II trial (23) showed that patients with liver metastases had significantly worse clinical response rates to treatment (either pembrolizumab alone or pembrolizumab + RT) than patients without liver metastases (**Supplementary Figure 1**).

Collectively, liver metastasis bodes poorly for patient survival or treatment response. Existing therapeutic regiments have proven ineffective, and new strategies are therefore needed to improve antitumor immunity and increase response rates in patients with liver metastases.

## Low-Dose Radiotherapy (LDRT)

Similar to immune-oncology agents, LDRT is capable of reprogramming the tumor microenvironment (TME), facilitating the infiltration of effector immune cells, and modulating the stroma in favor of tumor eradication. This has been borne out by a growing number of studies over the past decade, prompting much interest in the increasingly evident benefits of LDRT in the context of cancer immunotherapy (6–11, 24). Early evidence in a mouse model of localized neuroendocrine pancreatic tumors suggested that LDRT can remodel the TME in a variety of ways. LDRT has been shown to induce M1 macrophage polarization, leading to the production of cytokines/chemokines, such as IL-12, IFNγ, and RANTES. These attracted effector T cells and induced normalization of the tumor vasculature (24).

Our recent work builds upon the work of Klug and colleagues, confirming that LDRT polarizes pro-tumor M2-macrophages to the antitumor M1-phenotype, enhances the infiltration of CD4^+^ T cells and NK cells, and downregulates TGF-β inhibitory cytokine (6). In another study, we conducted proteomic analysis to evaluate the upregulation of TME-specific cytokines and stimulatory factors following LDRT. We found upregulation of Granzyme B, MIP1α, and CD137 (4-1BB) in tumor-infiltrating CD4^+^ T cells, indicating activation and effector functions (11). LDRT further augmented the efficacy of CPIs such as anti-CTLA-4 and anti-PD1 in murine lung adenocarcinoma models, as evidenced by reduced tumor growth and significantly prolonged survival.

Another advantage of LDRT is that it can readily be paired with more conventional high-dose radiotherapy (HDRT). HDRT can be directed to a primary tumor to release neoantigens and prime T cells (25), while LDRT can be administered to secondary metastatic lesions to modulate their stroma and create a welcoming environment for the responding T cells and NK cells, a novel combinatorial modality that we call the RadScopal™ technique (6). The efficacy of this technique is illustrated in another preclinical study, wherein a single-dose of HDRT (22 Gy), followed by four daily LD fractions (4 × 0.5 Gy), so called “postablation modulation (PAM)”, improved both local tumor control and remote lung metastases by reducing Tregs and M2 macrophages, hence enabling the infiltration of effector T cells into lung and breast carcinomas (8). Of note, this novel non-ablative regimen is safe and comes at minimal additional cost to that incurred by HDRT (9). The safety and efficacy of RadScopal™ treatment has been further validated by our recently published phase II trial of HDRT – with or without LDRT – for metastatic disease after progression on immunotherapy (9). This study showed that RadScopal™ therapy increased the response rate to 55%, compared to an 11% abscopal (HDRT alone) response rate, without added toxicity (3% RadScopal *vs*. 5% HDRT, grade ≥3 toxicity); while the RadScopal™ response rate for checkpoint-resistant liver lesions was even higher at 71% by our (unpublished) *post-hoc* analysis.

Recently, a randomized phase II trial of combined PD-L1 and CTLA-4 inhibitors with targeted LDRT (0.5 Gy per fraction) or hypofractionated radiation (HFRT, 3 fractions of 24 Gy) in patients with metastatic colorectal cancer revealed that both LDRT and HFRT impacted the local immune microenvironment and systemic immunogenicity (10). Once again, LDRT was found to increase the M1-to-M2 macrophage ratio and was associated with increased CD8^+^ T cell infiltration. Notably, the favorable enhanced M1/M2 ratio was not found in HFRT patients, illustrating the unique value of LDRT (10). Another elegant study by Herrera and colleagues confirmed our findings and supported the rationale for the combination of LDRT with immunotherapy in metastatic ovarian cancer. In this phase I clinical trial, LDRT paired with immunotherapy promoted CD4^+^ T cell infiltration, induced *de novo* inflammation, and promoted tumor regression in an IFNγ-dependent manner (7), thus supporting the rationale for combining LDRT with immunotherapy in tumors with low T cell infiltration.

## Low-Dose Radiotherapy (LDRT) for Liver Metastases

**Figure 1:**
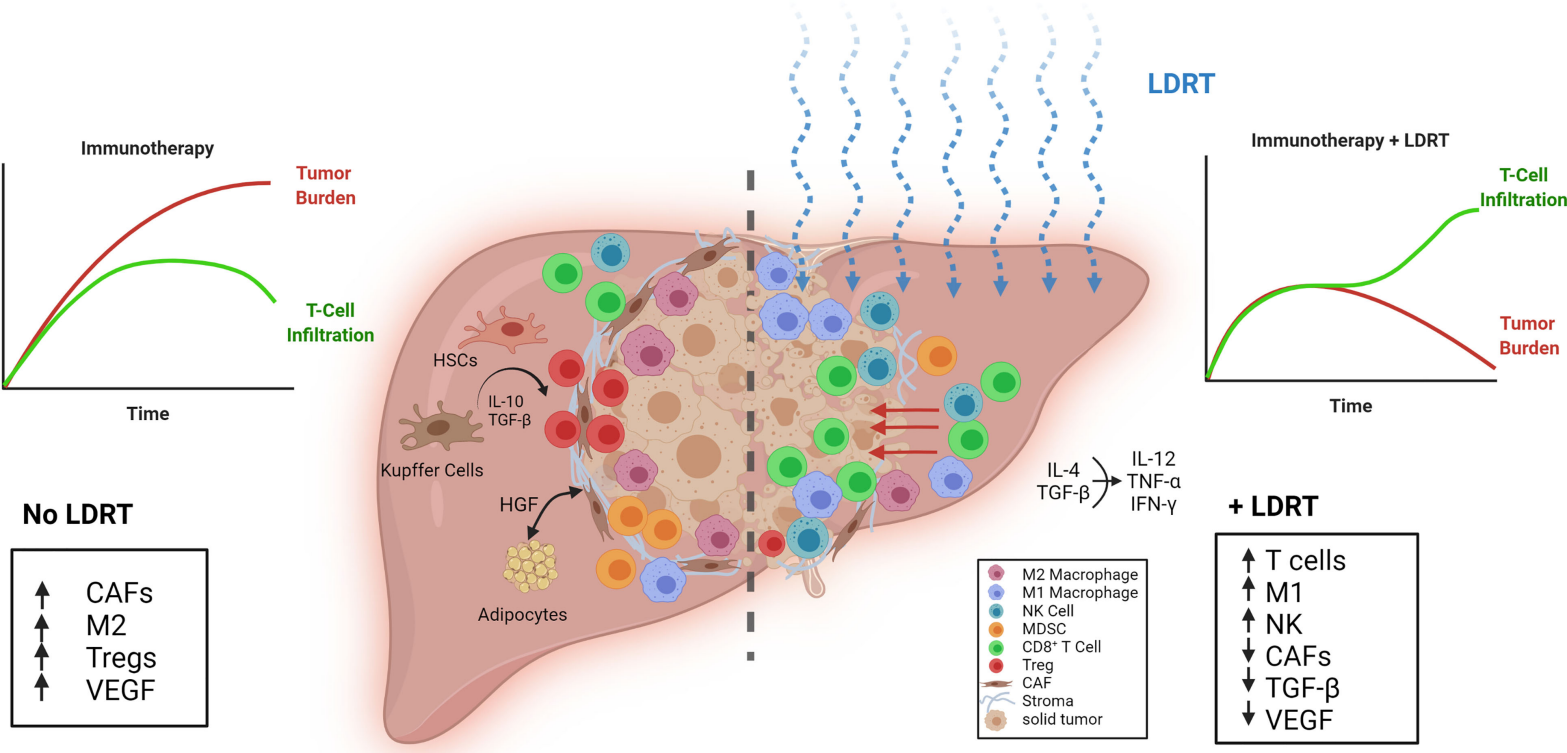
Reprogramming the tumor stroma by LDRT in liver metastases. (Left) The efficacy of immunotherapy is limited by unfavorable conditions in liver metastatic tumors such as a dense stroma, a low ratio of M1-to-M2 macrophages, increased TGF-β, vascular endothelial growth factor (VEGF) and cancer associated fibroblasts (CAFs). Liver resident cells, Kupffer cells, monocytic myeloid-derived suppressor cells (mMDSCs), and hepatic stellate cells (HSCs) promote Treg expansion through IL-10 and TGF-β release. (Right) Effect of low-dose radiotherapy (LDRT) on the immunosuppressive tumor microenvironment. LDRT repolarizes macrophages, decreases CAFs, and reduces TGF-β and VEGF. T cells and NK cells infiltrate the tumor through the disrupted stroma and receive positive stimulation from M1 macrophages.

The liver is an immune-privileged site, in that it can tolerate foreign antigens without invoking acute inflammatory responses (26). This immune quiescence makes the liver fertile soil for metastatic seeds to take root. Once embedded, tumors begin “terraforming” the liver, making it even more hospitable for them. Crosstalk between tumor and hepatic cells drives fibrosis through pro-fibrogenic interleukins production (IL-6, IL-8), integrin expression, and collagen deposition, which, together, enshroud the tumor in a stiff, protective barrier that shields it from immune surveillance and clearance (27–31). An overview of the immunostimulatory and inhibitory factors that drive outcomes in the hepatic TME is depicted in **Figure 1**. Specifically, the crosstalk between the hepatic and metastatic environments without LDRT (Left portion) and with LDRT (Right portion) is illustrated.

Other liver-resident cells such as Kupffer cells (KCs), hepatic stellate cells (HSCs), monocytic myeloid-derived suppressor cells (mMDSCs), and liver sinusoidal endothelial cells (LSECs) also contribute to T cell inactivation in the liver, as they secrete IL-10 and TGF-β that neutralize T cells and NK cells, promote T regulatory cells (Tregs), and polarize macrophages to pro-tumorigenic M2 phenotype (1). KCs are resident phagocytes of the liver that help protect and clear the liver of bacterial infections (32). However, in the presence of cancer, KCs may help shuttle tumor cells from circulation into the liver (1). The normally quiescent HSCs can be activated by KC factors such as TGF-β (1). Hepatocytes, LSECs, KCs, HSCs and dendritic cells can present antigens to recruited cells. However, antigen presentation by these cells can preferentially lead to immune tolerance rather than activation *via* expression of PD-L1 and engagement of PD1 on T cells, leading to T cell exhaustion and yet more production of immunosuppressive molecules such as IL-10 or TGF-β (1, 26, 33, 34). The direct impact of LDRT on KCs warrants further investigation.

TGF-β induces M2 macrophage polarization and production of vascular endothelial growth factor (VEGF) to promote tumor angiogenesis (1). Macrophages play a substantial role in driving tumor outcomes. Stromal macrophages limit CD8^+^ T cell infiltration and migration (35). Recently, it was reported that liver metastases can recruit immunosuppressive macrophages that actively promote apoptosis in antigen-specific T cells, further raising their profile as potential therapeutic targets (36). Accordingly, the same study found that liver-directed RT (8 Gy/1 fraction) decreased intrahepatic myeloid cells, coinciding with reduced CD8^+^ T cell apoptosis. This, when paired with anti-PD-L1, increased IFNγ production, CD4^+^ and CD8^+^ T cell proliferation, tumor regression, and overall survival.

As discussed above, homing of effector immunocytes such as T cells and NK cells to the tumor is increased following LDRT (6). This may partly result from the observed reduction in cancer-associated fibroblasts (CAFs) after LDRT (by >50%) (37). LDRT, by breaking the stroma barrier and modulating the TME, can enhance effector T cells activity and persistence that is required for successful CPI and ACT. A recent study showed that the activation of NK cells can be suppressed by HSCs (38). Thus, an NK cell-stimulating treatment such as IL-15 may further augment the antitumor effect of LDRT to liver metastases. Preliminary *post-hoc* analysis of our ongoing Phase-II trial (NCT02710253) (9), showed a lesion-specific response rate after LDRT that was higher for liver metastases (71%, n=7) compared to lung metastases (29% in lung, n=17).

**Figure 2:**
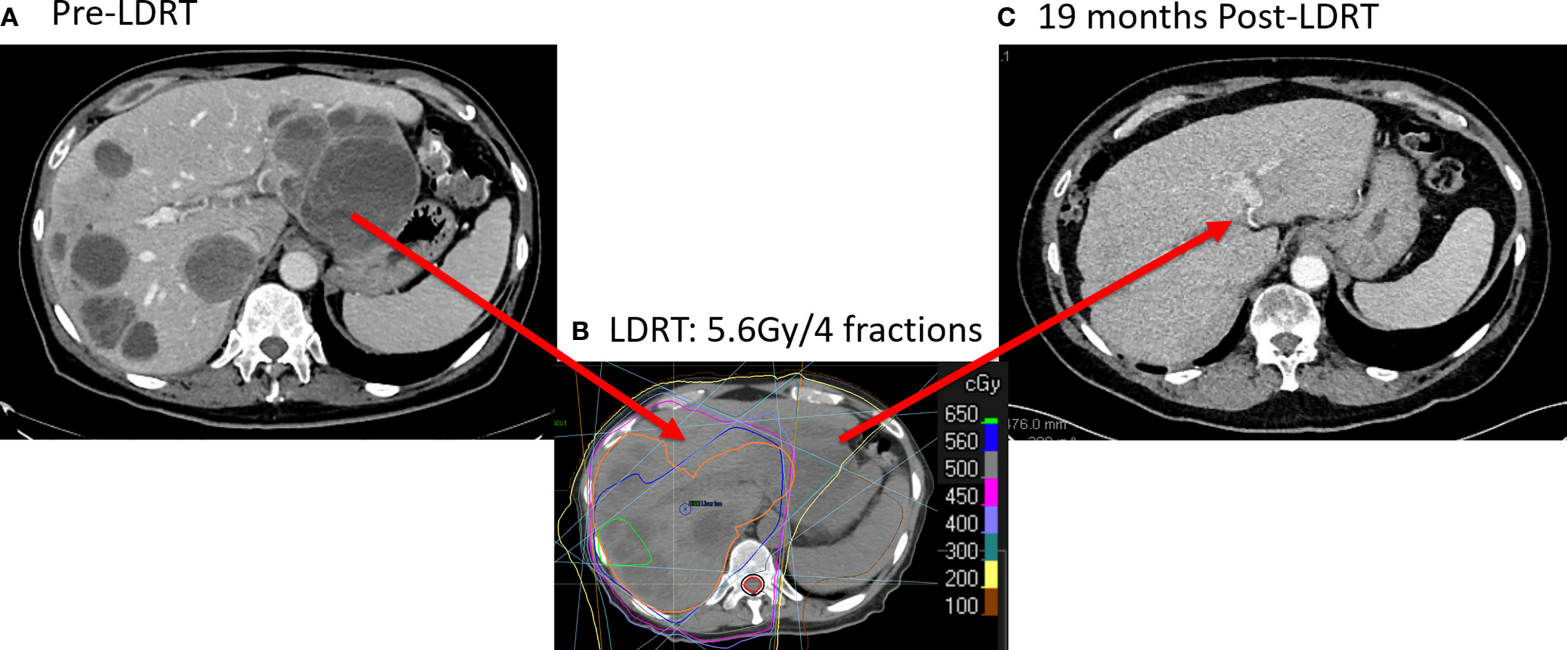
Complete Response with LDRT to Liver metastases. **(A)** CT scanning (9/4/2019) before LDRT showed multiple liver metastases. **(B)** The patient received 50 Gy/4 fractions to a lung lesion and 5.6 Gy/4 fractions to nearly the entire liver from 10/8/2019 to 10/11/2019. **(C)** 19 months after LDRT, CT scans (4/19/2020) showed a complete response in the liver.

We recently reported a representative case to illustrate these observations (11). The patient in the study presented with stage-IV melanoma with multiple metastases in liver, lung, bone and brain, which had progressed 3 months after T cell therapy and 1 month after resuming ipilimumab + nivolumab (**Figure 2A**). He received 4 fractions of 12.5 Gy (50 Gy in total) to a lung lesion, and 4 fractions of 1.4 Gy (totaling 5.6 Gy) to nearly the entire liver (**Figure 2B**). Four-months later, the patient achieved a partial response in liver. No changes in liver function or hepatic/pulmonary toxicity were noted. Two years after liver radiation treatment, the patient has no evidence of disease, with a durable and complete response for the liver metastases (**Figure 2C**).

## Conclusion and Future Directions

Pre-clinical and clinical data indicate that the immune suppressive environment in the liver is a major contributor for the lack of response of liver metastases to immunotherapy. Evidence from animal models and in patients with liver metastases support the notion that liver-directed LDRT could convert this environment into an immune-active state by reprogramming the stroma, which subsequently translates into improved responses to CPI. Another potential use of LDRT in liver metastasis disease is the combination with adoptive cell therapy (ACT), such as chimeric antigen receptor T-cell therapy (CAR-T) and engineered T-cell receptor therapy (TCR-T). One hurdle that ACT currently faces is the inhibitory stroma of solid tumors that is rich with immunosuppressive M2 macrophages, TGF-β and CAFs, which together limit cell-therapy infiltration and efficacy. Local delivery of LDRT could induce a higher M1/M2 ratio, decrease TGF-β and reduce CAFs, which results in enhanced T cell penetration and antitumor activity (37). Our ongoing preclinical studies (LDRT in combination with an anti-EGFR CAR-T) and clinical trial (NCT03132922) support the safety and antitumor potency of LDRT plus ACT in multiple solid tumors. More experimental studies based on liver metastatic models are needed to disclose liver-specific mechanisms of LDRT, and randomized clinical trials are required to support this novel strategy to get into common clinic applications.

## Author Contributions

Writing the manuscript: KH, HB, and VV. Reviewing and proofing the manuscript: GB, CL, EK, EL, EP, and TV. Making the figure: KH, EH, and HB. Supervision and oversight: MC and JW. All authors contributed to the article and approved the submitted version.

## Funding

This work was funded by the National Cancer Institute (*via* Cancer Center Support Core Grant P30CA016672 to The University of Texas MD Anderson Cancer Center).

## Conflict of Interest

JW reports grants from Varian Medical Systems, Bristol-Meyers Squibb; personal fees and other from Alpine Immune Sciences, Legion Healthcare Partners, Molecular Match, Nanorobotix, OncoResponse, and RefleXion Medical; grants and personal fees from Nanobiotics; grants, personal fees or equity from Checkmate Pharmaceuticals.

The remaining authors declare that the research was conducted in the absence of any commercial or financial relationships that could be construed as a potential conflict of interest.

## Publisher’s Note

All claims expressed in this article are solely those of the authors and do not necessarily represent those of their affiliated organizations, or those of the publisher, the editors and the reviewers. Any product that may be evaluated in this article, or claim that may be made by its manufacturer, is not guaranteed or endorsed by the publisher.
