# Supplementary material for: Novel Use of Low-Dose Radiotherapy to Modulate the Tumor Microenvironment of Liver Metastases
Source: Front Immunol. 2021 Dec 15;12:812210. doi: 10.3389/fimmu.2021.812210 (PMC8714746; doi:10.3389/fimmu.2021.812210)
Supplement: Supplementary file 1 [file DataSheet_1.docx]

Supplementary Material

**Supplementary Table 1.** Randomized Trials, currently enrolling, for Patients with Liver Metastases

| Trial | Modality | Setting | Randomization | Primary Endpoint |
| --- | --- | --- | --- | --- |
| NCT02738606 | Surgery | Colorectal cancer | Resection vs. no resection | 3-year OS |
| NCT03398291 | Surgery | Pancreatic cancer | Resection of primary + oligometastases vs. no resection | 2-year OS |
| NCT02597348 | Surgery | Colorectal cancer with unresectable metastases | Liver transplantation vs. no transplantation | 5-year OS |
| NCT04898504 | Surgery, HAI | Colorectal cancer progressed on first-line chemotherapy | 2^nd^ line chemotherapy alone vs. transplantation vs. HAI | 2-year OS |
| NCT03500874 | HAI | Colorectal cancer with resected liver metastases | HAI vs. no HAI | 3-year RFS |
| NCT02494973 | HAI | Colorectal cancer with resected liver metastases | HAI vs. no HAI | 3-year RFS, 1.5-year hepatic RFS |
| NCT02102789 | HAI | Colorectal cancer with unresectable liver metastases | HAI vs. no HAI | Rate of complete resection |
| NCT01785316 | HAI | Uveal melanoma | HAI vs. no HAI | 2-year OS |
| NCT03164655 | HAI | Colorectal cancer with unresectable liver metastases | HAI vs. no HAI | Rate of R0/R1 resection/ablation |
| NCT02885753 | HAI | Colorectal cancer; all receive panitumumab or bevacizumab with chemotherapy | HAI vs. no HAI | 2-year hepatic PFS |
| NCT02724540 | Chemoembolization | Neuroendocrine tumors | Bland embolization vs. TACE | 2-year hepatic PFS |
| NCT02936388 | Chemoembolization, radioembolization | Uveal melanoma | TACE vs. Y-90 | 1-year PFS |
| NCT03590119 | Radioembolization | Neuroendocrine tumors | Lu-177-dotatate intra-arterial vs. intra-venous | Post-treatment activity concentration between tumor and non-tumor |
| NCT03841305 | Embolization | Colorectal cancer | Portal vein embolization vs. portal & hepatic vein embolization | Volume of future remnant liver |
| NCT04832776 | Systemic therapy | Right-sided colon cancer; all receive FOLFOXIRI | Cetuximab vs. bevacizumab | 8-week objective response rate |
| NCT03493048 | Systemic therapy | Colorectal cancer with unresectable liver metastases | Cetuximab + FOLFOXIRI vs. cetuximab + FOLFOX | 6-month overall response rate |
| NCT02162563 | Systemic therapy | Colorectal cancer with unresectable liver metastases | RAS/BRAF wild-type & left-sided tumors (all receive FOLFOX/FOLFIRI): bevacizumab vs. panitumumab  RAS/BRAF mutated and/or right-sided (all receive bevacizumab): FOLFOX/FOLFIRI+vs. FOLFOXIRI | 2-year PFS |
| NCT04126655 | Systemic therapy | Colorectal cancer | Calciumfolinate vs arfolitixorin | Thymidylate synthase inhibition capacity |
| NCT03654131 | Radiotherapy, microwave ablation | Colorectal cancer with unresectable liver metastases | SBRT vs. microwave ablation | 3-year FFLP |
| NCT03135652 | Radiotherapy | Colorectal cancer with resected or ablated liver metastases | SBRT vs. no SBRT | 2-year DFS |
| NCT04079049 | Radiotherapy, ablation, surgery | Breast cancer | Systemic therapy alone vs. resection/ablation/SBRT | 3-year OS |
| NCT03458975 | Other | Any cancer | Sonoporation vs. no sonoporation | 2-month objective response rate |
| NCT03477019 | Other | Breast and colorectal cancers | Focused ultrasound vs. systemic therapy alone | 10-12 week objective response rate |
| NCT03428477 | Other | Colorectal cancer with resected liver metastases | EPA vs. placebo | 2-year PFS |
| NCT03326791 | Other | Colorectal cancer | Low-dose aspirin vs. placebo | 3-year DFS |

Abbreviations: OS, overall survival; HAI, hepatic artery infusion; RFS, recurrence-free survival; PFS, progression-free survival; TACE, transarterial chemoembolization; SBRT, stereotactic body radiation therapy; FFLP, freedom from local progression; DFS, disease-free survival; EPA, eicosapentanoic acid.

**
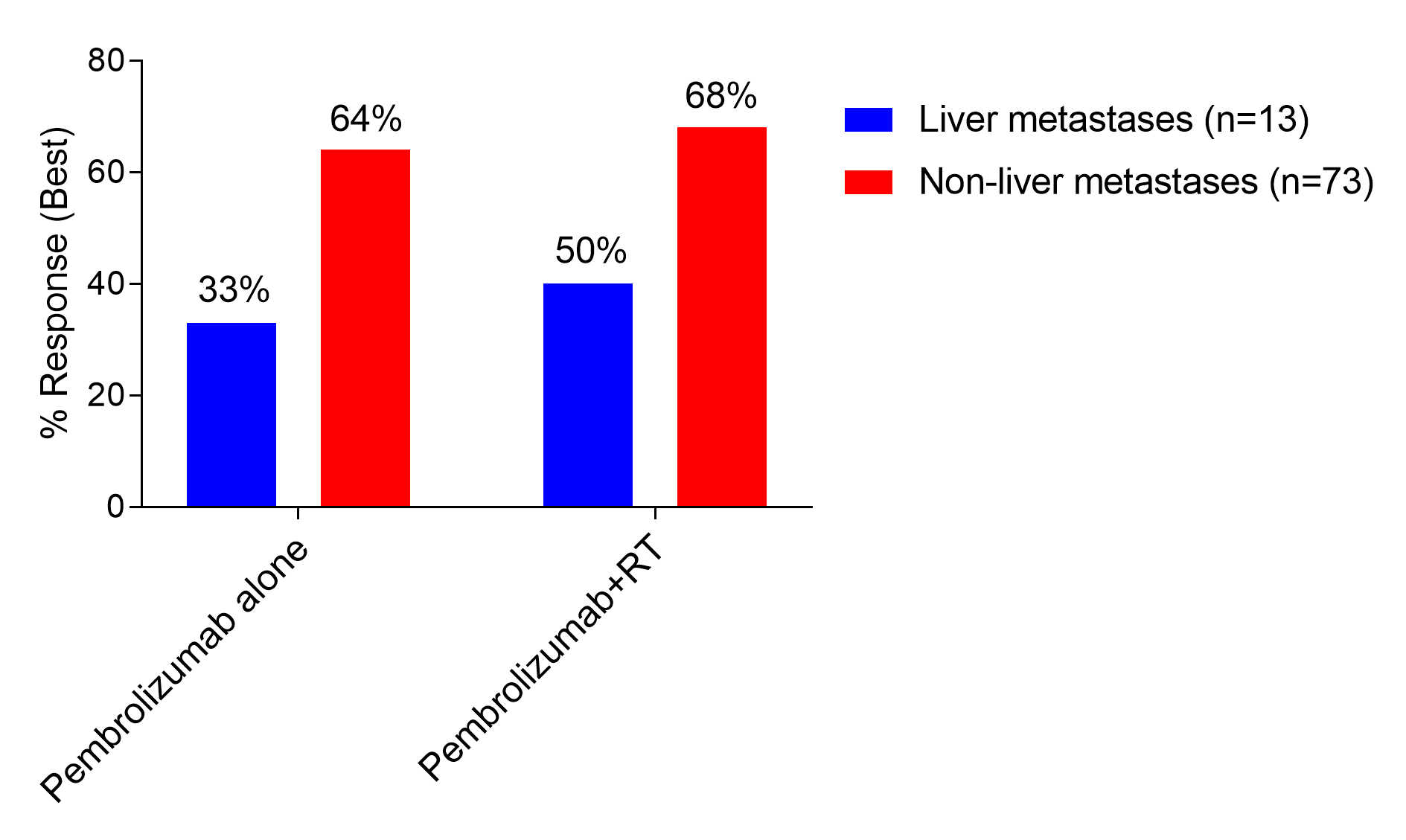
**

**Supplementary Figure 1.** Clinical response to pembrolizumab with or without RT in advanced NSCLC with or without liver metastases. In this phase I/II trial, patients with metastatic NSCLC were treated with pembrolizumab alone or pembrolizumab concurrent with RT for lung and liver lesions. The choice of RT included SBRT (50 Gy in 4 fractions) if clinically feasible or traditionally fractionated RT (45 Gy in 15 fractions) if not. This figure shows the rates of best clinical response to pembrolizumab with or without RT based on liver metastases or not by a post-hoc analysis. NSCLC, non-small cell lung cancer; RT, radiotherapy; SBRT, stereotactic body radiotherapy.
